# Next-Generation Sequencing Technology Combined With Multiplex Polymerase Chain Reaction as a Powerful Detection and Semiquantitative Method for Herpes Simplex Virus Type 1 in Adult Encephalitis: A Case Report

**DOI:** 10.3389/fmed.2022.905350

**Published:** 2022-06-15

**Authors:** Weibi Chen, Yingfeng Wu, Yan Zhang

**Affiliations:** Xuanwu Hospital, Capital Medical University, Beijing, China

**Keywords:** encephalitis, herpes simplex virus type 1, metagenomic next-generation sequencing, multiplex PCR, sequencing

## Abstract

**Background:**

Traditional testing for specific microbes or categories of central nervous system (CNS) infectious diseases is often limited in sensitivity and timeliness. However, failure to initiate a timely etiological diagnosis and corresponding treatment in patients with neurologic infections contribute to poor outcomes.

**Case Summary:**

A 58 year-old male presented acutely with fever, abnormal mental behavior, seizures and decreased consciousness. Brain magnetic resonance imaging (MRI) showed an abnormal FLAIR/T2 signal mainly in the left thalamus, temporal lobe, insular lobe, and bilateral hippocampus. To identify the pathogen, the cerebrospinal fluid (CSF) sample of the patient was used for metagenomic next-generation sequencing (mNGS) analysis and multiplex polymerase chain reaction (mPCR). The results showed 188 herpes simplex virus (HSV-1)-specific sequences. After acyclovir and foscarnet sodium treatment, the ratio of HSV-1/internal reference reads decreased from 813/493 to 695/1961, which coincided with clinical remission.

**Conclusion:**

This study indicates that mNGS combined with mPCR may be an effective method for etiological diagnostic and dynamic clinical surveillance for HSV-1 encephalitis.

## Introduction

Encephalitis is defined as the presence of an inflammatory process of the brain in association with clinical evidence of neurologic dysfunction. As an important cause of morbidity, mortality, and permanent neurological sequelae, encephalitis remains a worldwide health problem. Of the pathogens reported to cause encephalitis, the majority are viruses (1). Viral agents include primary neurotropic viruses such as arboviruses, herpesviruses, and rabies virus, as well as other nervous system pathogens such as enteroviruses, measles, respiratory viruses, etc., causing disease in the central nervous system (CNS) and elsewhere in the body. However, the pathogens for encephalitis cases are not identified in approximately 50% of patients (1, 2). Fever, headache, altered mental status, seizures, and/or focal neurologic signs, are common but non-specific symptoms of encephalitis, that overlap with those of different viral encephalitis, non-viral infectious entities or inflammatory encephalitis of non-infectious origin (3). These symptoms do not reliably identify the underlying etiology. In addition, metabolic or toxic encephalopathy, can also mimic viral encephalitis. Since empirical treatment may be ineffective or even harmful, accurate information about etiological agents and individualized management of a patient who presents with encephalitis are required to ensure good outcomes.

Traditional diagnostic techniques (e.g., virus culture, hemagglutination inhibition assay, enzyme immunoassay, and direct fluorescent antibody detection) were once the mainstays for pathogen detection, but the sensitivity and timeliness are limited for viral pathogens. Advances in molecular technology have now allowed its use as a clinical diagnostic tool. Metagenomic next-generation sequencing (mNGS) provides an unbiased analysis method, that can theoretically identify viruses, bacteria, parasites, fungi, and other pathogens by sequencing the total RNA or DNA in the samples of patients with known sequences (4–7). Previous studies have reported that mNGS of cerebrospinal fluid (CSF) obtained from patients with CNS infectious diseases can effectively identify different pathogens (5, 8), but none of these studies indicated mNGS as a semiquantitative method in clinical application.

Here, we report a case of herpes simplex encephalitis (HSE). In the case, mNGS analysis and multiplex PCR (mPCR) were used to identify the herpes simplex virus (HSV-1) and served a semiquantitative method to determine the pathogenic load.

## Case Description

**FIGURE 1:**
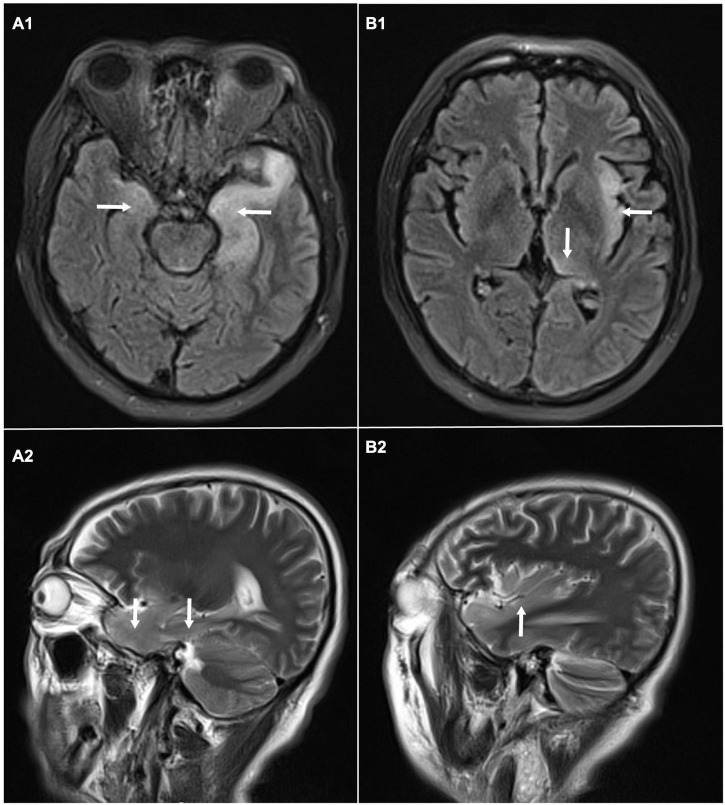
Brain magnetic resonance imaging: abnormal signal of T2-weighted images and fluid-attenuated inversion recovery images (T2/FLAIR) in the left thalamus, temporal lobe, insular lobe and bilateral hippocampus. **(A1)** Axial MRI showed high signals on FLAIR in the left temporal lobe and bilateral hippocampus. **(A2)** Sagittal MRI showed high signals on T2 in the temporal lobe and hippocampus. **(B1)** Axial MRI showed high signals on FLAIR in left thalamus, and insular lobe. **(B2)** Sagittal MRI showed high signals on T2 in left insular lobe.

**FIGURE 2:**
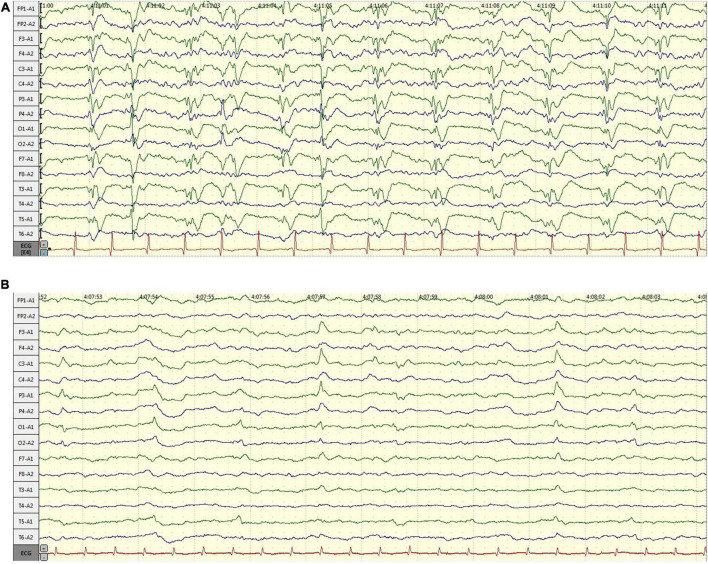
The changes of electroencephalogram (EEG) over time. **(A)** EEG performed on admission: lateralized periodic discharges (PDs) on the left; **(B)** EEG performed 7 days after admission: PDs had disappeared.

A 58-year-old male, with a history of hypertension, was admitted because of fever, abnormal mental behavior, epileptic seizures, and decreased consciousness. In the morning before admission, he was found to be slow to respond with a mild fever of 37.6°C. Blood examinations showed a peripheral blood leukocyte count of 15,860/mm^3^ (lymphocytes: 12.1%); C-reactive protein level of 25.4 mg/L; and normal procalcitonin level. Brain magnetic resonance imaging (MRI) revealed an abnormal signal of T2-weighted images and fluid-attenuated inversion recovery images (T2/FLAIR) in the left thalamus, temporal lobe, insular lobe and bilateral hippocampus (Figure 1). During that day, he had frequent seizures and gradually felt increasingly sleepy. Four days before admission, the patient experienced anorexia and abdominal distension with no anal discharge. A computed tomography (CT) scan showed luminal distension in the proximal part of the intestine and accumulation of luminal contents. Physical examination on admission revealed stupor with a positive meningeal irritation sign, moist rales in both lungs, abdominal distension and hyperactive bowel sounds (at least 10 times/min). Further diagnostic work-up was performed. Lumbar puncture was performed on the second day after admission, which showed elevated intracranial pressure of 260 mmH_2_O. Cerebrospinal fluid (CSF) analysis revealed inflammatory changes with pleocytosis of 24 leukocytes/μl (96% monocytes) and normal biochemistry with a glucose level of 55.62 mg/dl and a protein level of 33 mg/dl. CSF culture was also performed for pathogen detection, which did not reveal any pathogens. An electroencephalogram (EEG) performed on admission showed lateralized periodic discharges (LPDs) on the left (Figure 2A). The presumptive diagnosis of viral encephalitis was made. However, serological tests for infectious agents, including herpes simplex virus (HSV-1, 2), varicella zoster virus (VZV), cytomegalovirus (CMV), Epstein–Barr virus (EBV), and human herpes virus (HHV6, 7, 8), were all negative. The CSF sample was then sent for mNGS analysis and mPCR to identify the pathogen, which was approved by the Ethics Committee of Xuanwu Hospital, Capital Medical University [No. (2020) 104]. Written informed consent was provided by the patient. The mPCR procedure was performed as described below.

### Nucleic Acid Extraction

The CSF samples were centrifuged at 13,000 rpm for 10 min and ground on a grinding mill (Tiss-24, Jingxin, Shanghai, China) at 60 Hz for 10 min. The ground samples were then used for DNA/RNA extraction and purification (Zymo BIOMICS DNA/RNA Miniprep Kit, R2002) according to the manufacturer’s instructions.

### Construction of the Sequencing Library

The extracted nucleic acids were used to construct the pathogen-targeted high-throughput sequencing library. The library was built by using a Pathogeno One High-Throughput Sequencing Library Construction kit (Shanghai Pathogeno Medical Technology Co., Ltd., China, SJ0005). A group of nucleic acid standards with known concentrations were added to the amplification system. In this process, two rounds of PCR were conducted. The sample nucleic acid and cDNA were used as templates, and a total of 524 microorganism-specific primers were chosen for multiple PCR amplification to enrich the target pathogen sequences, which contain bacteria (294), viruses (79), fungi (65), parasites (38), spirochetes (7), and others (41). Following the amplification step, PCR products were purified by beads and then amplified using primers with sequencing adapters and different barcodes. After purification of the final amplified products by agarose gel electrophoresis, quality inspection, and quantification were performed using a Qubit4.0 fluorometer. Normally, the library fragment size was approximately 400 bp, and the library concentration was at least 1 ng/μl.

### High-Throughput Sequencing

The concentration of the mixed library was requantified and then diluted to a final concentration of 4 nM. Next, 5 μl of the mixed library was added with 5 μl of freshly prepared NaOH (0.2 M). After vortexing and centrifuging at 280 g for 1 min, the library was placed at room temperature for 5 min. The denatured library was sequenced on a MiSeq system (Illumina, Inc., San Diego, CA, United States) using a MiSeq reagent kit v2 (average 0.05 million reads per library, with sequencing read length = PE60). FastQ files were generated by MiSeq Reporter software.

### Data Analysis

The raw data were first identified by the linker, the reads with a paired-end length > 60 bp were retained, and then low-quality filtering was performed to retain reads with Q30 > 50% as high-quality data. The paired-ended aligned reads were compared with the pathogen database to confirm the number of sequences (reads) in each sample. Through the statistical analysis of the read number after sequencing, we can obtain the ratio between the read number of the specific amplification target and the read number of these standards, and then calculated the approximate content of the specific amplification target.

**FIGURE 3:**
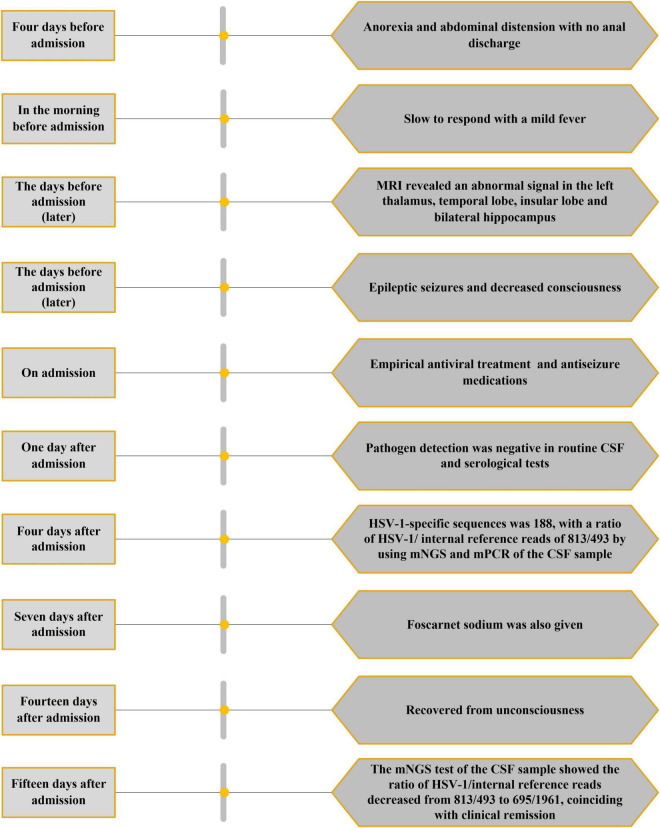
Timeline of the clinical history.

The CSF sequencing results returned 3 days later, showing that the number of HSV-1-specific sequences was 188, with a ratio of HSV-1/internal reference reads of 813/493. On admission, empirical antiviral treatment (acyclovir: 10 mg/kg intravenously every 8 h) and antiseizure medications (intramuscular injection of phenobarbital 0.2 g followed by phenobarbital (90 mg) orally every 8 h and levetiracetam (1,500 mg) orally every 12 h were given to the patient. However, he remained unconscious 7 days after admission, when the plasma concentration of phenobarbital was 28.49 μg/ml. An EEG was then performed again, which indicated that periodic discharge had disappeared, and only a small amount of epileptic discharges could be seen, as shown in Figure 2B. In case of the possibility of acyclovir resistance, foscarnet sodium (50 mg/kg) was also given intravenously twice per day. Fourteen days after admission, the patient recovered from unconsciousness. A repeated CSF examination was performed, which showed a fewer inflammatory changes with pleocytosis of 12 leukocytes/μl (100% monocytes), a glucose level of 66 mg/dl, and a protein level of 42 mg/dl. The mNGS test of the CSF sample showed a ratio of HSV-1/internal reference reads of 695/1961. Compared with the first mNGS analysis, the relative pathogenic load was reduced five times, which coincided with clinical remission. In addition, tests for anti-HSV IgM and IgG in serum as well as anti-HSV IgG in CSF were positive. Since the virus load determined *via* mNGS did not drop to zero, antiviral therapy (intravenous drip of acyclovir and foscarnet sodium) was continued for another 2 weeks. A summary of the timeline is presented in Figure 3.

The follow-up results at 6 months after discharge were as follows: self-care, no epilepsy or emotional abnormalities, mild cognitive impairment, and normal character orientation. The patient could communicate with people, but he often forgot some words and some people’s names.

## Discussion

In this case, we used mNGS and mPCR to identify the pathogen in the CSF of the patient, and we found that the specific sequences mapped to HSV-1 genomic regions and that the relative pathogenic load was reduced five times, which coincided with the improvement in clinical symptoms.

CNS infectious diseases are caused by different pathogens. The detection of a wide range of pathogens is essential to maximize the diagnostic rate for patients with CNS infectious diseases (9). However, the causative pathogens for encephalitis cannot be identified in some cases (1, 2), in part due to a lack of standardized diagnostic approaches, while the traditional microbiological tests (culture, smear, and immunoassay) chosen are often pathogen specific. Specific etiology diagnosis is important to guide the corresponding therapy and avoid unnecessary or even potential harm to patients (10). Under these circumstances, mNGS as a broad-spectrum pathogen analysis method, has revolutionized the field of infectious diseases, especially given the limited CSF samples. In recent years, mNGS has been successfully used to identify viral (11–15), bacterial (16–18), Ureaplasma parvum (19), fungal (20), toxoplasmic (21), and tuberculous (8) pathogens in CNS infections. A previous multicenter study in the United States reported that mNGS could detect pathogens (13 of 58) that were not detected by conventional methods (5). Compared with that of traditional clinical diagnosis, mNGS techniques also dramatically reduced the diagnostic period to less than 3 days, as seen in this study.

Currently, there are no reliable criteria or standard analysis methods to accurately discriminate between insignificant contaminants and true infectious organisms, or to define a positive mNGS result without the need for a confirmatory test. Based on a prospective multicenter study, Xing et al. proposed that different CNS infectious diseases were associated with different positive diagnostic criteria due to variations in lifestyles and genomic sequences (8). The pathogen HSV-1 identified in this study is consistent with the clinical manifestations of herpes simplex encephalitis, and the improvement in clinical symptoms after corresponding antiviral treatment verified the accuracy of the etiological diagnosis. Recently, some criteria have been proposed, such as mapping of at least three sequencing reads to three different genomic regions of a virus genome or the absence of virus sequencing reads in negative controls (5, 22).

In this study, the level of pathogens reads relative to the internal reference in the two CSF samples of the patient was calculated by using mNGS combined with mPCR. Intriguingly, the decrease in the relative level of HSV-1 coincided with the improvement in clinical symptoms. Using an internal reference as a benchmark, the relative level of the virus can be accurately detected and objectively interpreted even if the level of the virus is low. Therefore, compared with the traditional qualitative detection of mNGS, our semiquantitative detection method offers a better sensitivity for pathogen identification and pathogenic load determination.

According to the guidelines, empirical antibiotics are commonly initiated in patients with suspected encephalitis, pending the results of diagnostic studies. Early administration of acyclovir for 14–21 days was recommended by the Infectious Diseases Society of America (23). With the application of antiviral drugs, the mortality of HSE has decreased (24). However, acyclovir-resistant herpes encephalitis and relapse of HSV encephalitis after completion of acyclovir therapy have been reported (25). In this patient, in the case of acyclovir resistance, foscarnet sodium was also given. Although significant clinical improvement was observed in the patient after 2 weeks of antiviral therapy, the viral load in the CSF had not yet decreased to zero, antiviral drugs were therefore continued for another 2 weeks to prevent relapse, which is a much longer course than recommended. At the follow-up 6 months after discharge, the patient’s condition was relatively good and satisfactory.

## Conclusion

This study proves that mNGS combined with mPCR may be an effective method for etiological diagnosis and dynamic clinical surveillance for HSV-1 encephalitis.

## Ethics Statement

Written informed consent was obtained from the patient for the publication of this case report and any potentially identifiable images or data included in this article.

## Author Contributions

WC designed, administrated the study, analyzed the data, and drafted the manuscript. YW provided the resources and participated within the analysis. YZ provided the resources, supervised the study, and reviewed the manuscript. All authors contributed to the article and approved the submitted version.

## Conflict of Interest

The authors declare that the research was conducted in the absence of any commercial or financial relationships that could be construed as a potential conflict of interest.

## Publisher’s Note

All claims expressed in this article are solely those of the authors and do not necessarily represent those of their affiliated organizations, or those of the publisher, the editors and the reviewers. Any product that may be evaluated in this article, or claim that may be made by its manufacturer, is not guaranteed or endorsed by the publisher.
